# P-360. Real-world Effectiveness and Safety Outcomes in People with HIV-1 Switching to Dolutegravir + Lamivudine (DTG + 3TC) with Unknown Prior Genotype: a Systematic Literature Review and Meta-analysis

**DOI:** 10.1093/ofid/ofaf695.578

**Published:** 2026-01-11

**Authors:** John Boulos, Jeremy Fraysse, Eva Fernvik, Bryn Jones, Gustavo Verdier, Julie Priest, Emilio Letang

**Affiliations:** ViiV Healthcare, Apex, NC; ViiV Healthcare, Apex, NC; ViiV Healthcare, Apex, NC; ViiV Healthcare, Apex, NC; ViiV Healthcare, Montréal, QC, Canada, Pointe-Claire, Quebec, Canada; ViiV Healthcare, Apex, NC; ViiV Healthcare, Madrid, Spain, Madrid, Madrid, Spain

## Abstract

**Background:**

In phase 3 interventional studies, DTG/3TC demonstrated durable efficacy as a switch option for people with HIV-1 with virologic suppression and no pre-existing resistance-associated mutations (RAMs) or prior virologic failures (VFs). Historical or baseline genotypes are not always available in clinical practice. In a pooled TANGO/SALSA post hoc analysis, high virologic suppression (VS) rates were maintained after switching to DTG/3TC regardless of availability of prior genotype. Previous studies suggest that switching to DTG + 3TC with known pre-existing M184V/I may not impact effectiveness. We evaluate real-world effectiveness and impact of RAMs in people with HIV-1 switching to DTG + 3TC with unknown prior genotype.

**Methods:**

A systematic literature review (SLR) was conducted, searching databases and relevant congresses reporting effectiveness and safety outcomes for people switching to DTG + 3TC with unknown prior genotype (published 1/2013-11/2024). Meta-analysis feasibility was conducted and appropriate. Based on SLR data, single-arm meta-analyses will produce point estimates for proportions of individuals with VF across studies.

**Results:**

Of 310 publications identified representing 61,334 people with HIV-1 using DTG + 3TC, 13 reported outcomes for 3402 unique individuals switching to DTG + 3TC with unknown prior genotype. Eleven studies (n=3380) reported effectiveness outcomes at 6 months to 96 weeks (∼24 months). High VS rates (96%-100%) were maintained after switch. Overall, 0.30% (10/3380) of individuals experienced VF or discontinued for virologic reasons. M184V was detected at VF in 0.06% (2/3380) of individuals, and 1 person had T97A, E138K, and N155H, which confers low-level resistance to DTG, at treatment discontinuation (0.03%; 1/3380; HIV-1 RNA 540 copies/mL). In 5 studies reporting safety and/or tolerability outcomes at 30 weeks to 36 months (n=356), 0.84% (3/356) of individuals discontinued DTG + 3TC due to adverse events unrelated to study drug. Meta-analysis point estimates are forthcoming.

**Conclusion:**

Consistent with interventional studies, DTG + 3TC was effective and well tolerated, with rare emergence of M184V/I or integrase RAMs, among people with HIV-1 switching to DTG + 3TC with unknown prior genotype in clinical practice.

**Disclosures:**

John Boulos, PharmD, GSK: Stocks/Bonds (Public Company)|ViiV Healthcare: Employee Jeremy Fraysse, MS, GSK: Stocks/Bonds (Public Company)|ViiV Healthcare: Stocks/Bonds (Private Company) Eva Fernvik, PhD, GSK: Stocks/Bonds (Public Company)|ViiV Healthcare: Stocks/Bonds (Private Company) Bryn Jones, MBChB, GSK: Stocks/Bonds (Public Company)|ViiV Healthcare: Employee Gustavo Verdier, BSc, BPharm, MBA, ViiV Healthcare: Employee Julie Priest, MSPH, GSK: Stocks/Bonds (Public Company)|ViiV Healthcare: Employee Emilio Letang, MD, MPH, PhD, GSK: Stocks/Bonds (Public Company)|ViiV Healthcare: Employee

